# Clinical Outcomes of Percutaneous Full-Endoscopic Interlaminar Lumbar Discectomy (PFEILD): A Retrospective Single-Surgeon Pilot Cohort Study

**DOI:** 10.7759/cureus.116744

**Published:** 2026-09-22

**Authors:** Asim Abdelgader A Farah, Mahdi Bassi, Hassan Siddiq, Nihal Abdelrazig A Mohammed, Thorea Abdelgadir A Farah, Rana Alrasheed, Omaim Hussein

**Affiliations:** 1 Orthopaedic Surgery, Dr. Soliman Fakeeh Hospital, Jeddah, SAU; 2 Orthopaedic Surgery, Qassim Health Cluster, Buraydah, SAU; 3 Diabetes and Endocrinology, Qassim Health Cluster, Buraydah, SAU; 4 Applied Medical Sciences, Taibah University, Al-Ula, SAU

**Keywords:** full endoscopic lumbar discectomy, interlaminar approach, lumbar disc herniation, macnab criteria, minimally invasive spine surgery, oswestry disability index, radiculopathy, visual analog scale

## Abstract

Introduction

Low back pain (LBP) is a major health problem among adults, with lumbar disc herniation (LDH) being a common underlying cause. While many patients with LDH respond to conservative management, surgical intervention may be required in selected cases. Percutaneous full-endoscopic interlaminar lumbar discectomy (PFEILD) is a minimally invasive percutaneous endoscopic discectomy that offers several potential advantages, including reduced tissue disruption, shorter hospital stays, and faster postoperative recovery. However, few studies in our region have evaluated the clinical and functional outcomes of this technique.

Methods

A total of 25 patients who attended the spinal surgery unit at Dr. Soliman Fakeeh Hospital (DSFH) in Jeddah, Saudi Arabia, and underwent PFEILD between January 2019 and January 2023 were included. The study aimed to evaluate the outcomes of PFEILD in terms of length of hospital stay, time to return to work, postoperative complications, recurrence, and clinical and functional improvement. The patients were followed postoperatively for approximately 24-36 months. Clinical and functional outcomes were assessed using the MacNab criteria, the Visual Analog Scale (VAS) for leg pain, and the Oswestry Disability Index (ODI). Data were collected from the hospital electronic health record (EHR) and analyzed using IBM SPSS Statistics for Windows, Version 29 (Released 2022; IBM Corp., Armonk, New York, United States).

Results

Of the 25 patients, 68% (n=17) were discharged within one postoperative day, while 96% (n=24) experienced no postoperative complications. An excellent postoperative outcome according to the MacNab criteria was reported in 80% (n=20) of patients. Preoperatively, 92% (n=23) of participants had a VAS score for leg pain >5/10, compared with 96% (n=24) who had a postoperative VAS score <5/10. The difference between the preoperative and postoperative VAS scores was statistically significant (P<0.001). Furthermore, 72% (n=18) of patients had a preoperative ODI score >40, whereas none had a postoperative ODI score >40. The difference between preoperative and postoperative ODI scores was statistically significant (P<0.001).

Conclusion

Minimally invasive PFEILD demonstrated favorable clinical and functional outcomes, with significant improvements in leg pain and disability, short postoperative hospital stays, early return to work, and a low rate of complications. Further large-scale, prospective, multi-center studies are recommended to validate these findings and assess the long-term outcomes of this technique.

## Introduction

Low back pain (LBP) is one of the major causes of chronic incapacity in adults, and it affects 4% to 33% of the population at any given point [1]. Lumbar disc herniation (LDH) is the cause of 90% of neuropathic back pain, with a prevalence of 1 to 3% [2].

The causes and risk factors for LDH are fully explained in the literature [3-6]. LDH, characterized by the displacement of the nucleus pulposus beyond the limits of the disc space [7], results in intense pain in the lumbar region, changes in the strength, reflexes, and sensitivity of the affected patients [8,9].

Major advancements have been made in recent years regarding the diagnosis and treatment of low back pain due to LDH. In most cases, LBP and the associated radiating pain to the lower limbs are treated conservatively with anti-inflammatory medication, physiotherapy, and epidural steroid injection [2,10]. With these conservative methods, the pain usually resolves over a period of six weeks in 10 to 20% of cases [2,11]. However, in cases where radicular pain persists and becomes refractory to adequate conservative management, surgical intervention is warranted [11].

For symptomatic LDH, various surgical options exist, including conventional open discectomy, microdiscectomy, and percutaneous endoscopic discectomy. The choice between these options depends on many factors such as the availability of equipment and the surgeon’s experience and preference [12]. Open discectomy, first introduced by Mixter and Barr in 1934, is considered the gold standard for the treatment of symptomatic lumbar disc herniation [1,2,13,14]. However, because of the greater soft tissue damage during open surgery, various minimally invasive techniques have been developed [13,15]. The most used minimally invasive procedures are endoscopic transforaminal and translaminar discectomies, which have a success rate of 60 to 95% [14,16]. Minimally invasive percutaneous endoscopic discectomy has the advantage of avoiding large skin incisions, excessive soft tissue dissection, and bone damage. Additionally, they result in minimal blood loss, reduced hospital stays, and early return to work [17-24].

Compared with open surgery, minimally invasive percutaneous endoscopic discectomy offers several advantages, including reduced blood loss and complication rates, minimal soft-tissue damage, shorter operative time and hospital stays, faster recovery, and earlier return to work. The procedure can also be performed under local or epidural anesthesia, allowing intraoperative communication with the patient and real-time feedback that may help reduce the risk of nerve root injury [25]. Consistent with these potential benefits, previous studies have demonstrated comparable postoperative pain and functional outcomes between endoscopic and open approaches, while also reporting favorable clinical outcomes with the endoscopic technique, particularly in terms of pain relief and functional recovery [6,12,26-30].

Additionally, the minimally invasive percutaneous endoscopic discectomy is associated with minimal recurrence rate. However, literature varies in the rate of recurrence, which ranges from 1.2 to 13% [24,31-37]. Compared to other procedures, fully endoscopic discectomy is believed to be safe with a quick anatomical approach, which preserves soft tissue and bony anatomy, decreases the time of surgery and hospital stays, improves pain score and neurological symptoms with a faster return to work, but no single study that explored these factors in depth. Additionally, no study in our region has specifically studied this procedure and evaluated its outcome.

## Materials and methods

Study design and setting

This hospital-based retrospective pilot cohort study was conducted at the Spinal Surgery Unit of Dr. Soliman Fakeeh Hospital (DSFH), Jeddah, Saudi Arabia. Patients who underwent percutaneous full-endoscopic interlaminar lumbar discectomy (PFEILD) between January 2019 and January 2023 were evaluated.

Study objective

This study aimed to evaluate the initial clinical and functional outcomes of PFEILD in a Saudi Arabian tertiary care center, including changes in leg pain and disability, postoperative complications, recurrence, hospital stay, and time to return to work.

Participants

A total population sampling (census) technique was used. All patients who underwent PFEILD at DSFH between January 2019 and January 2023 were considered eligible for inclusion. A total of 25 patients met the study criteria and were included in the analysis. The inclusion and exclusion criteria are presented in Table 1.

**Table 1:** Inclusion and exclusion criteria of the study population PFEILD, percutaneous full-endoscopic interlaminar lumbar discectomy (PFEILD); DSFH, Dr. Soliman Fakeeh Hospital.

Inclusion Criteria	Exclusion Criteria
Patients who underwent PFEILD at DSFH between January 2019 and January 2023.	Patients who underwent other types of lumbar discectomy procedures.
Patients who had available preoperative and postoperative clinical data sufficient for outcome assessment.	Patients who had insufficient clinical or follow-up data for assessment of the study outcomes.
	Patients who underwent revision discectomy procedures.

Data collection and outcome measures

Data were extracted from electronic health records and included demographic characteristics, clinical history, preoperative findings, operative details, follow-up duration, and postoperative outcomes. Patients were followed postoperatively for approximately 24-36 months. Clinical outcomes were assessed using the Visual Analog Scale (VAS) for leg pain [38], Oswestry Disability Index (ODI) version AU2.1b [39-44], and modified MacNab criteria [45]. Additional postoperative outcomes included complications, recurrent disc herniation, revision surgery, length of hospital stay, operative time, and time to return to work.

Surgical technique

All procedures were performed under general anesthesia with the patient in the prone position on a Jackson operating table, with the abdomen free. The operative level was confirmed using intraoperative fluoroscopy. A paramedian interlaminar approach was performed from the clinically and radiologically symptomatic side through an approximately 8 mm skin incision.

A VERTEBRIS lumbar full-endoscopic system (RIWO Spine Endoscopic System; Richard Wolf GmbH, Knittlingen, Germany) was used. The system incorporates an 8 mm outer diameter access sheath with a working channel accommodating instruments up to 4.0 mm in diameter. Continuous normal-saline irrigation was maintained using the Fluid Control Arthro-Spine irrigation (Richard Wolf GmbH, Knittlingen, Germany) and suction system with the spine-mode software module.

After placement of the working sheath, the interlaminar window was assessed endoscopically. Limited laminotomy was performed when the interlaminar space was narrow, particularly at L4-L5. At L5-S1, additional bony decompression was generally unnecessary when an adequate interlaminar window was present. Partial medial facetectomy was performed when required to obtain adequate access in cases of narrow interlaminar windows, paracentral disc herniation, or lateral recess stenosis. When necessary, lateral bone resection was performed directly on the floor of the spinal canal within the working field.

The ligamentum flavum was resected in all cases to expose the epidural space. The affected nerve root was identified and protected throughout the procedure by controlled rotation of the beveled working sheath. The herniated disc fragment was removed using endoscopic grasping instruments until satisfactory nerve-root decompression was achieved. Final endoscopic inspection was performed to confirm adequate decompression and hemostasis.

Following removal of the endoscope and working sheath, the approximately 8 mm skin incision was closed with a single suture. Patients were mobilized early after recovery from anesthesia and were discharged according to their postoperative clinical condition and institutional protocol.

Statistical analysis

Data were collected, coded, and analyzed using IBM SPSS Statistics for Windows, Version 29 (Released 2022; IBM Corp., Armonk, New York, United States). Descriptive statistics were used to summarize the demographic, clinical, and operative characteristics of the study population. Categorical variables were presented as frequencies and percentages, whereas continuous variables were summarized using means and standard deviations (SD) or medians and ranges, as appropriate according to their distribution.

Because of the relatively small sample size, the distribution of the paired differences was assessed for approximate normality before applying the test. All statistical tests were considered statistically significant at a p-value<0.05.

Preoperative and postoperative VAS scores for leg pain were compared using a paired-samples t-test. The mean change in VAS score was reported together with its 95% confidence interval (CI). VAS scores were also categorized as <5 and ≥5, and changes in pain category between the preoperative and postoperative assessments were evaluated using McNemar’s test. Where the number of discordant pairs was small, the exact McNemar test was used.

Given the small sample size, statistical findings were interpreted with consideration of the reduced statistical power and increased uncertainty of the estimates.

Preoperative and postoperative ODI scores were compared using a paired-samples t-test to evaluate changes in functional disability following surgery. The mean difference between preoperative and postoperative ODI scores was reported with a 95% CI. A one-tailed paired-samples t-test was additionally used to assess the prespecified directional hypothesis that postoperative ODI scores would be lower than preoperative scores.

Modified MacNab outcomes, laterality of lower-limb pain, level of disc herniation, postoperative complications, and nationality were summarized using frequencies and percentages. Operative time, length of hospital stay, and time to return to work were summarized using appropriate descriptive statistics.

## Results

A total of 25 patients were included in this study, comprising 13 male subjects and 12 female subjects, with a nearly equal distribution between the two sexes (Figure 1).

**Figure 1:**
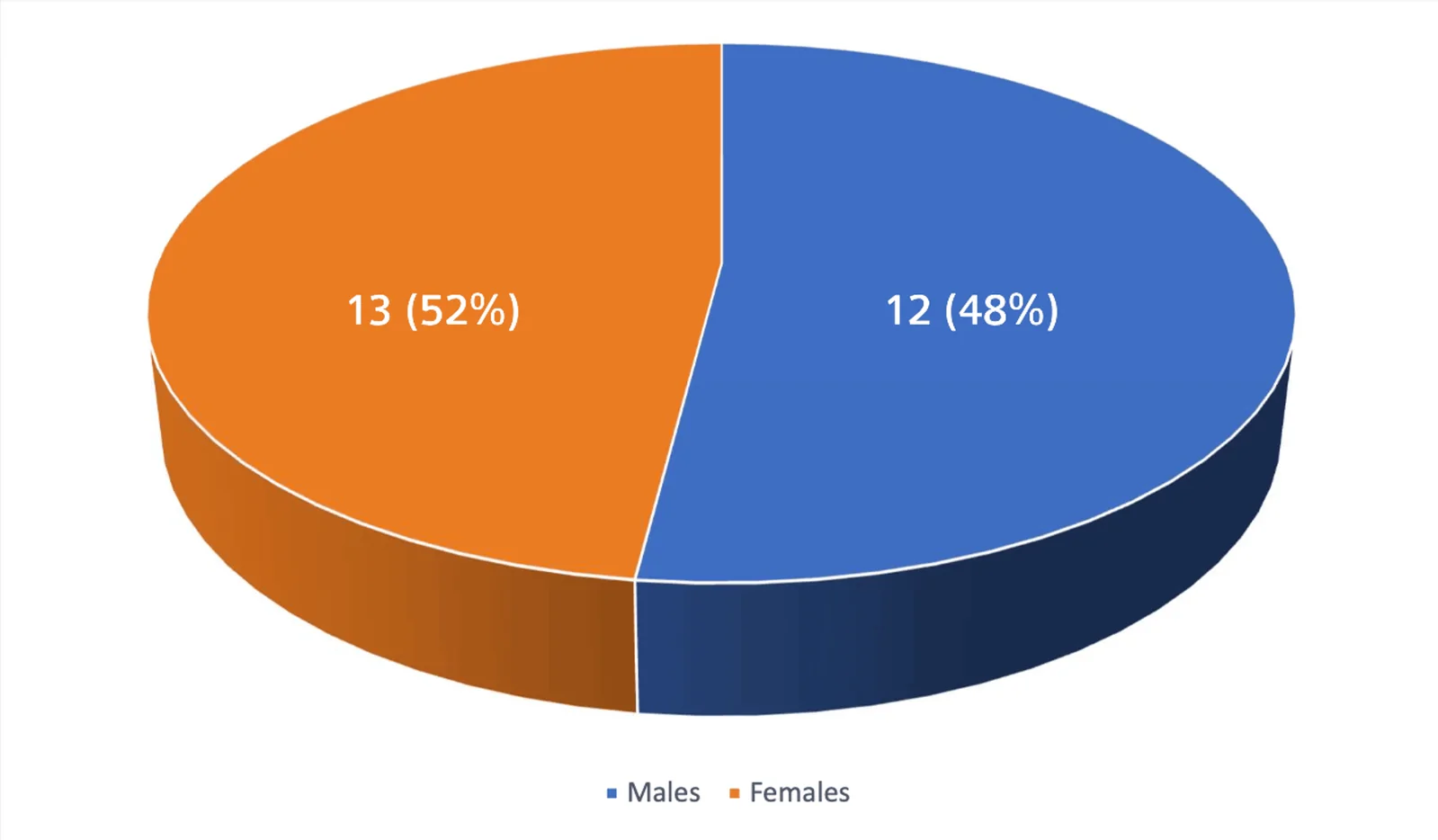
Sex distribution of the study population (n=25)

The mean age of the participants was 42.32 ± 15.79 years, with an age range of 20-78 years (Table 2).

**Table 2:** Descriptive statistics of age in the study population (n=25)

Statistics	Value
Mean	42.32
Median	37.00
Mode	30.00
Standard Deviation	15.79

Most participants were Saudi nationals (n=17), while the remaining eight participants represented eight different nationalities (Figure 2).

**Figure 2:**
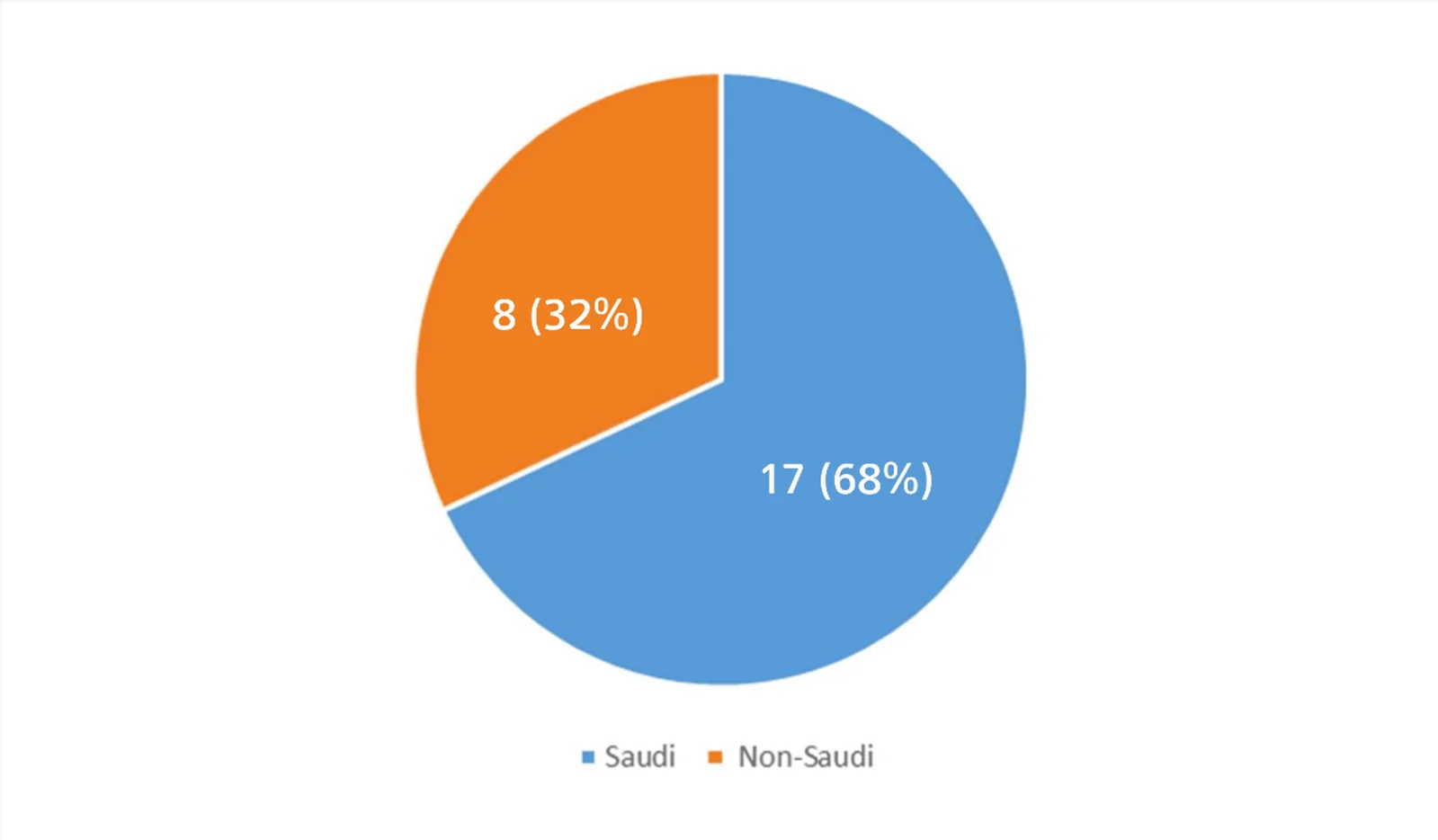
Nationality distribution of the study population (n=25) The figure illustrates the proportion of Saudi participants compared with the participants of other nationalities.

The postoperative follow-up period was approximately 24-36 months. Several outcome measures were used to assess pain relief and functional outcomes following PFEILD. At postoperative follow-up, the modified MacNab criteria demonstrated an excellent outcome in the majority of patients (80%, n=20), while none of the patients were classified as having a poor outcome (Figure 4).

**Figure 3:**
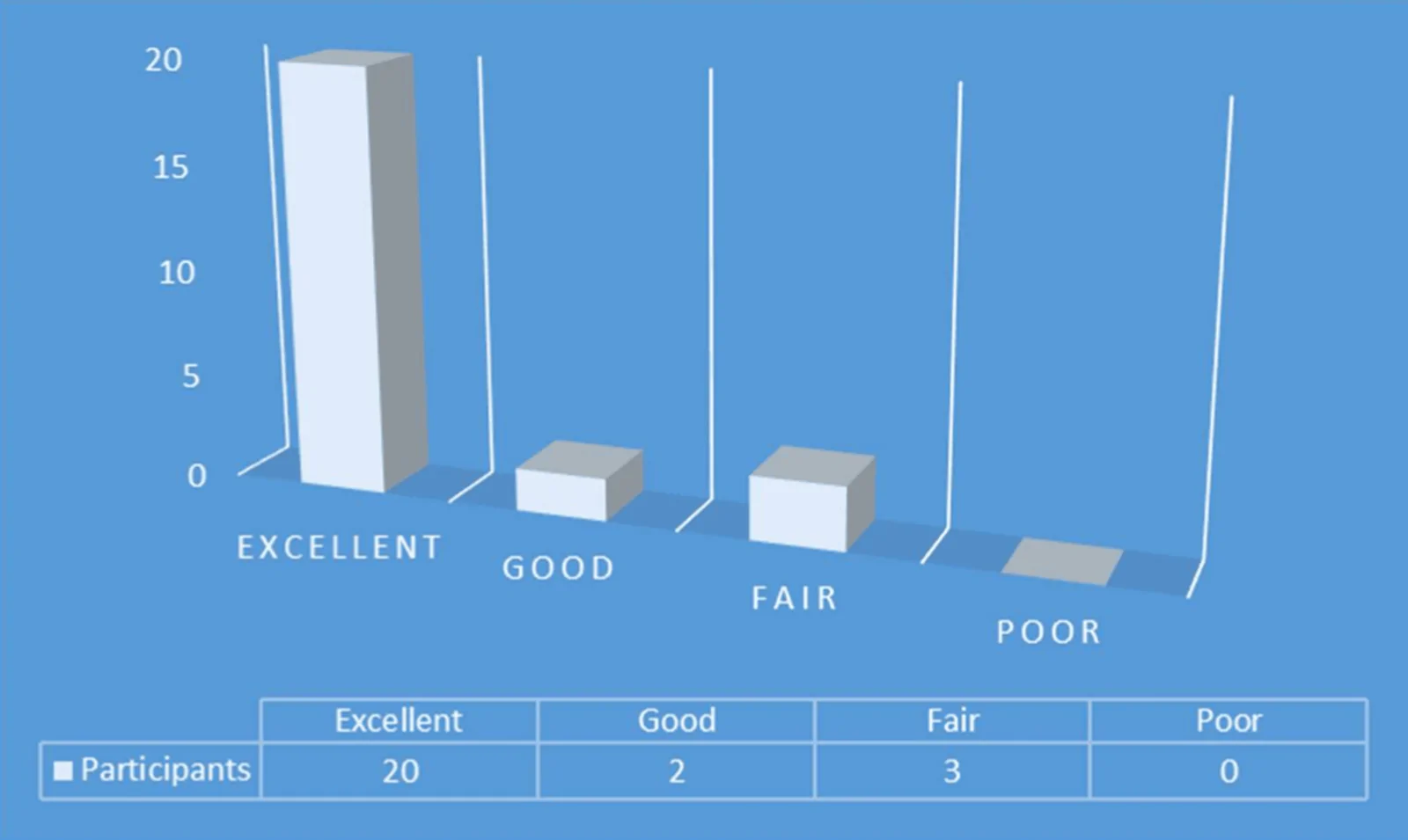
Postoperative clinical outcomes according to the modified MacNab criteria Excellent outcomes were observed in 20 patients (80%), with no patients classified as having a poor outcome.

Pain intensity before and after surgery was assessed using the VAS for leg pain. Preoperatively, the mean VAS score was 8.32 ± 1.80, with 23 patients (92%) reporting scores greater than 5/10, while two patients (8%) reported a score of 5/10 (Table 3).

**Table 3:** Preoperative VAS scores for leg pain Visual analog scale (VAS) scores represent preoperative leg pain intensity on a 0–10 scale. Values are presented as frequencies and percentages.

Score	Frequency	Percent
5/10	2	8.0
6/10	4	16.0
7/10	2	8.0
8/10	3	12.0
9/10	4	16.0
10/10	10	40.0
Total	25	100.0

Postoperatively, the mean VAS score decreased to 2.20 ± 1.58, with 24 patients (96%) reporting scores below 5/10 and only one patient (4%) reporting a score of 7/10 (Table 4 and Figure 4).

**Table 4:** Postoperative VAS scores for leg pain Visual analog scale (VAS) scores represent postoperative leg pain intensity on a 0–10 scale. Values are presented as frequencies and percentages.

Score	Frequency	Percent
0/10	2	8.0
1/10	8	32.0
2/10	6	24.0
3/10	4	16.0
4/10	4	16.0
7/10	1	4.0
Total	25	100.0

**Figure 4:**
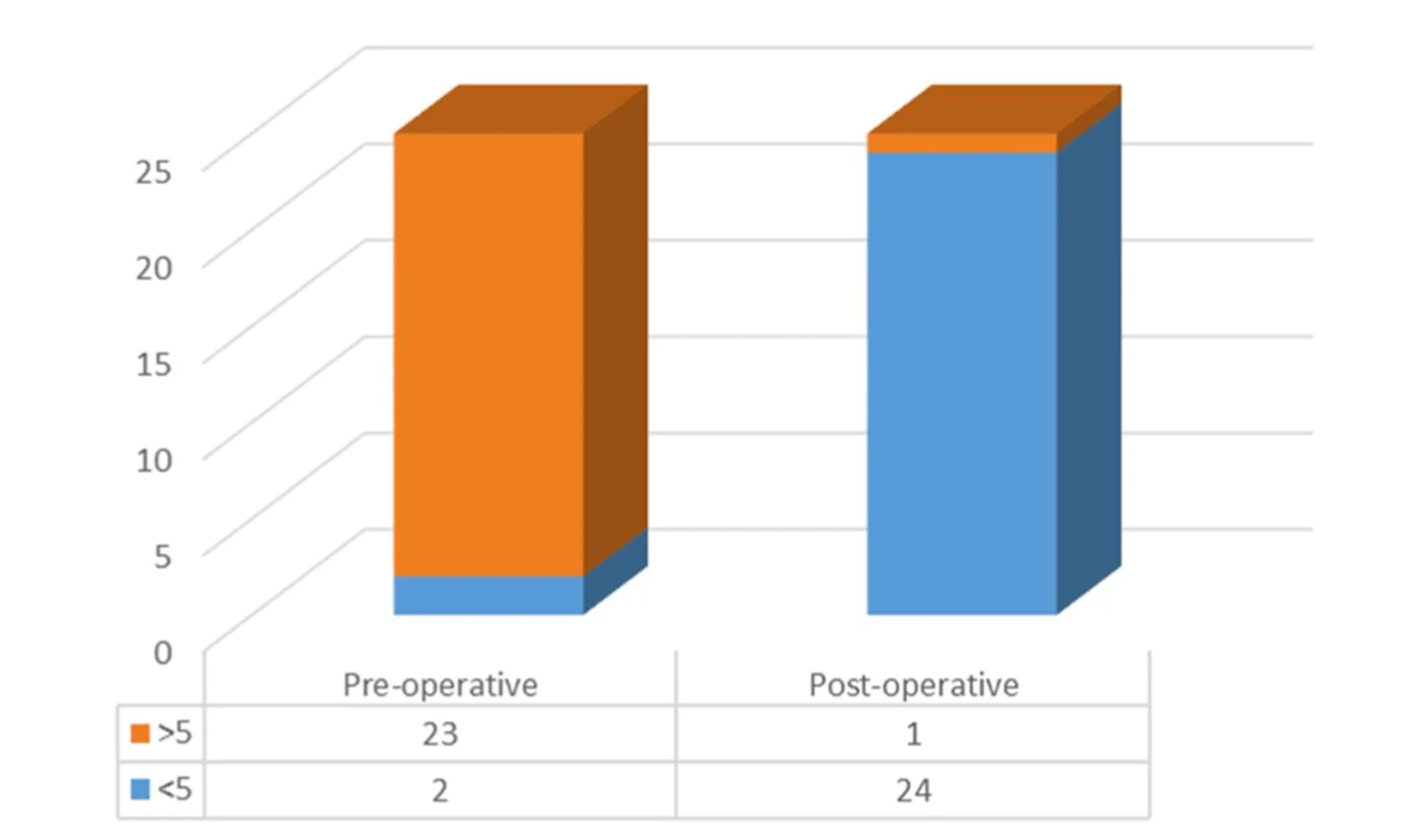
Comparison of preoperative and postoperative VAS scores for leg pain The figure illustrates the change in visual analog scale (VAS) scores for leg pain before and after surgery.

A statistically significant difference was observed between the preoperative and postoperative mean VAS scores using the paired t-test (Table 5).

**Table 5:** Comparison of preoperative and postoperative VAS scores for leg pain Values are presented as mean ± standard deviation. Preoperative and postoperative visual analog scale (VAS) scores were compared using a paired-samples t-test. A p-value <0.05 was considered statistically significant.

	Paired Differences					t	df	P value
		Std.	Std. Error Mean	95% Confidence Interval of the Difference				
	Mean	Deviation		Lower	Upper			
VAS preoperative - VAS postoperative	6.040	2.318	.464	5.083	6.997	13.028	24	<0.001

When VAS scores were dichotomized into two categories (≤5 and >5), a statistically significant change in pain category was also observed using the McNemar test (Table 6).

**Table 6:** Comparison of preoperative and postoperative VAS pain categories Visual analog scale (VAS) scores were categorized as ≤5 or >5. Preoperative and postoperative categories were compared using the McNemar test. A p-value <0.05 was considered statistically significant.

		VAS postoperative		Total	P value (two-sided)
		<5	>5		
VAS preoperative	≤5	2	0	2	<0.001
	>5	22	1	23	
Total		24	1	25	

Regarding postoperative hospital stay, most patients (68%, n=17) were discharged after one postoperative day, while only one patient had a hospital stay of approximately four days (Figure 5).

**Figure 5:**
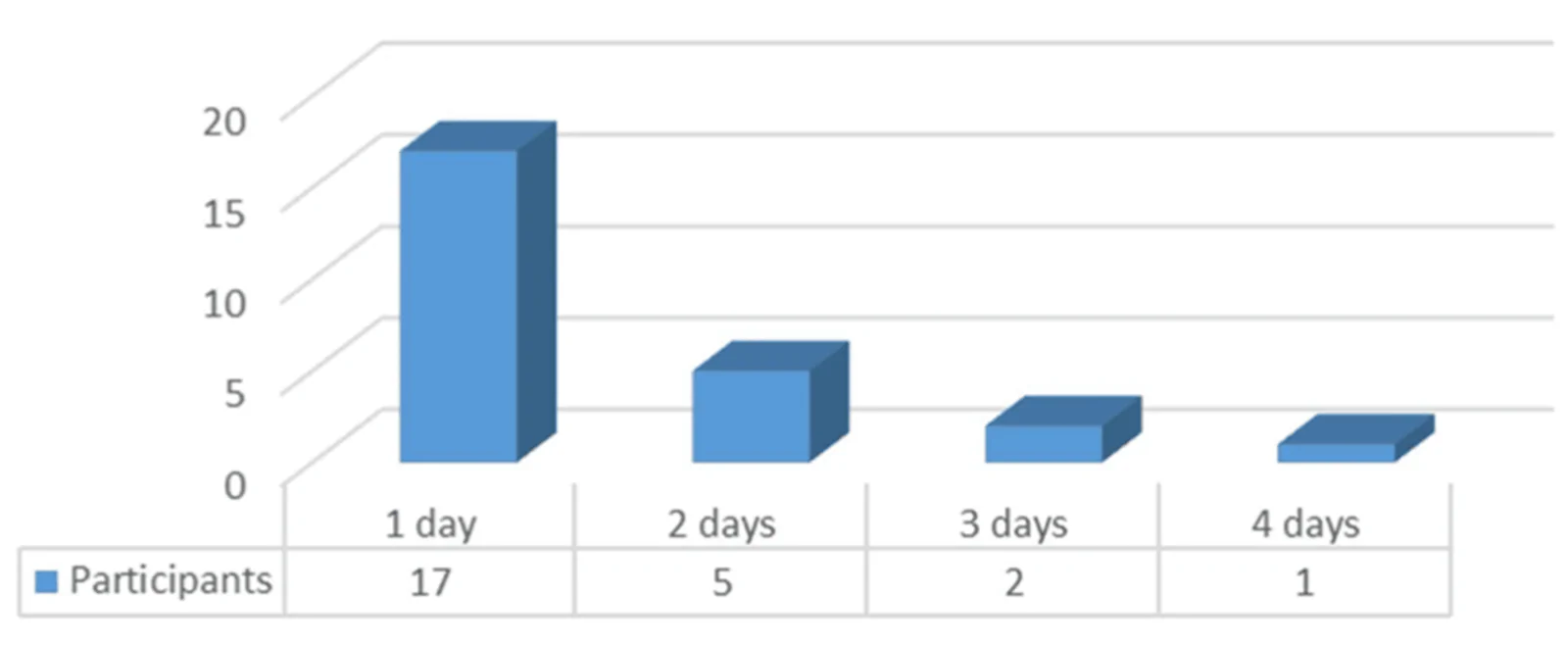
Postoperative length of hospital stay The figure illustrates the distribution of postoperative hospital stay in the days following surgery.

At the time of initial presentation, lower-limb pain was reported in the left leg in seven patients (28%), bilaterally in seven patients (28%), and in the right leg in 11 patients (44%) (Figure 6).

**Figure 6:**
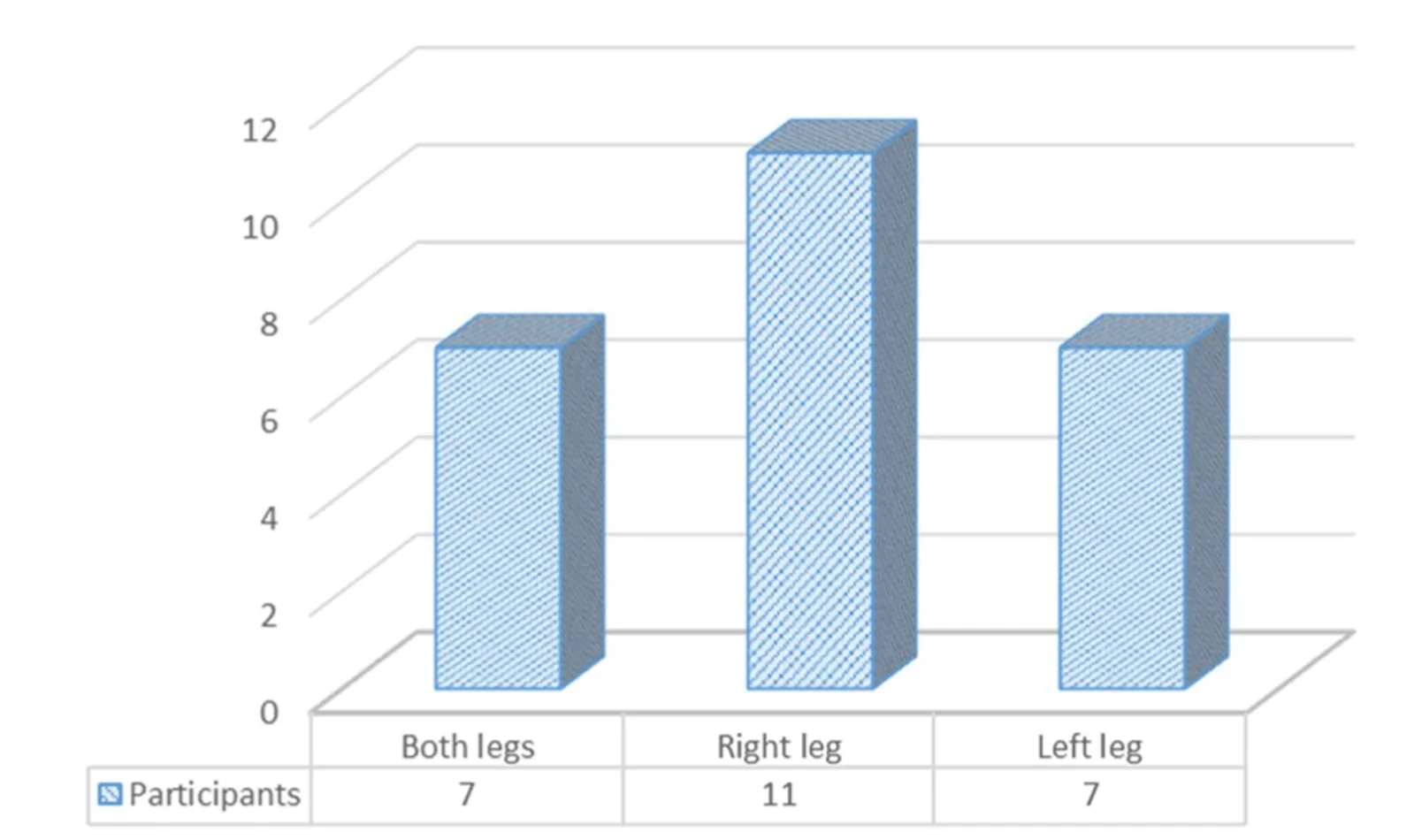
Laterality of lower-limb pain at the initial presentation The figure illustrates the distribution of lower-limb pain according to its laterality at initial presentation: right-sided, left-sided, or bilateral.

Regarding the level of disc herniation, more than half of the patients had an L5-S1 disc herniation (52%, n=13), making it the most frequently involved level. The L4-L5 level was the second most frequently involved level, while the remaining patients had disc herniations at other levels (Figure 7).

**Figure 7:**
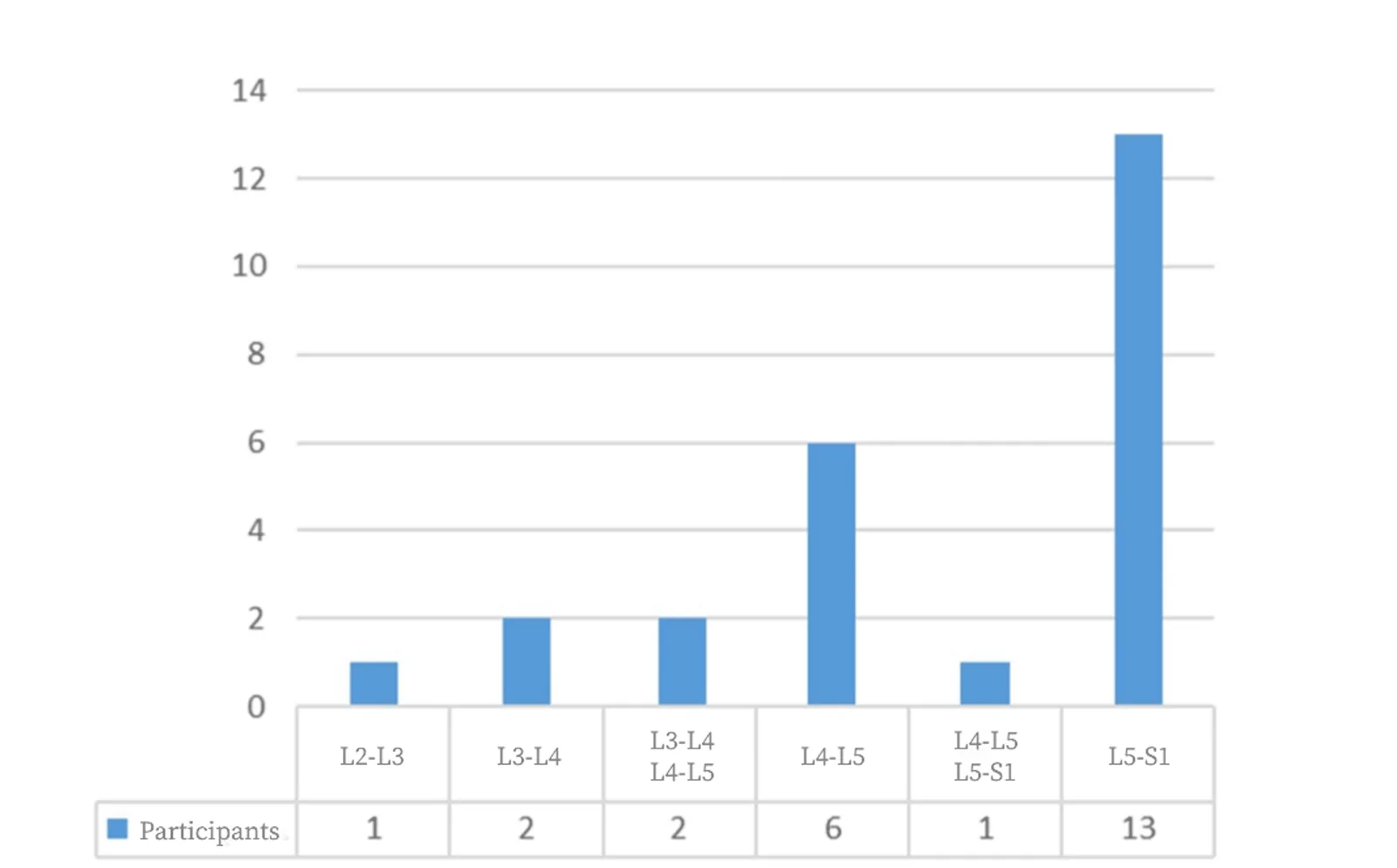
Distribution of disc herniation according to the spinal level The figure illustrates the distribution of lumbar disc herniations according to the affected spinal level, with L5–S1 representing the most frequently involved level.

Postoperative surgical complications were uncommon, occurring in only one patient, while 24 patients (96%) experienced no postoperative complications (Figure 8).

**Figure 8:**
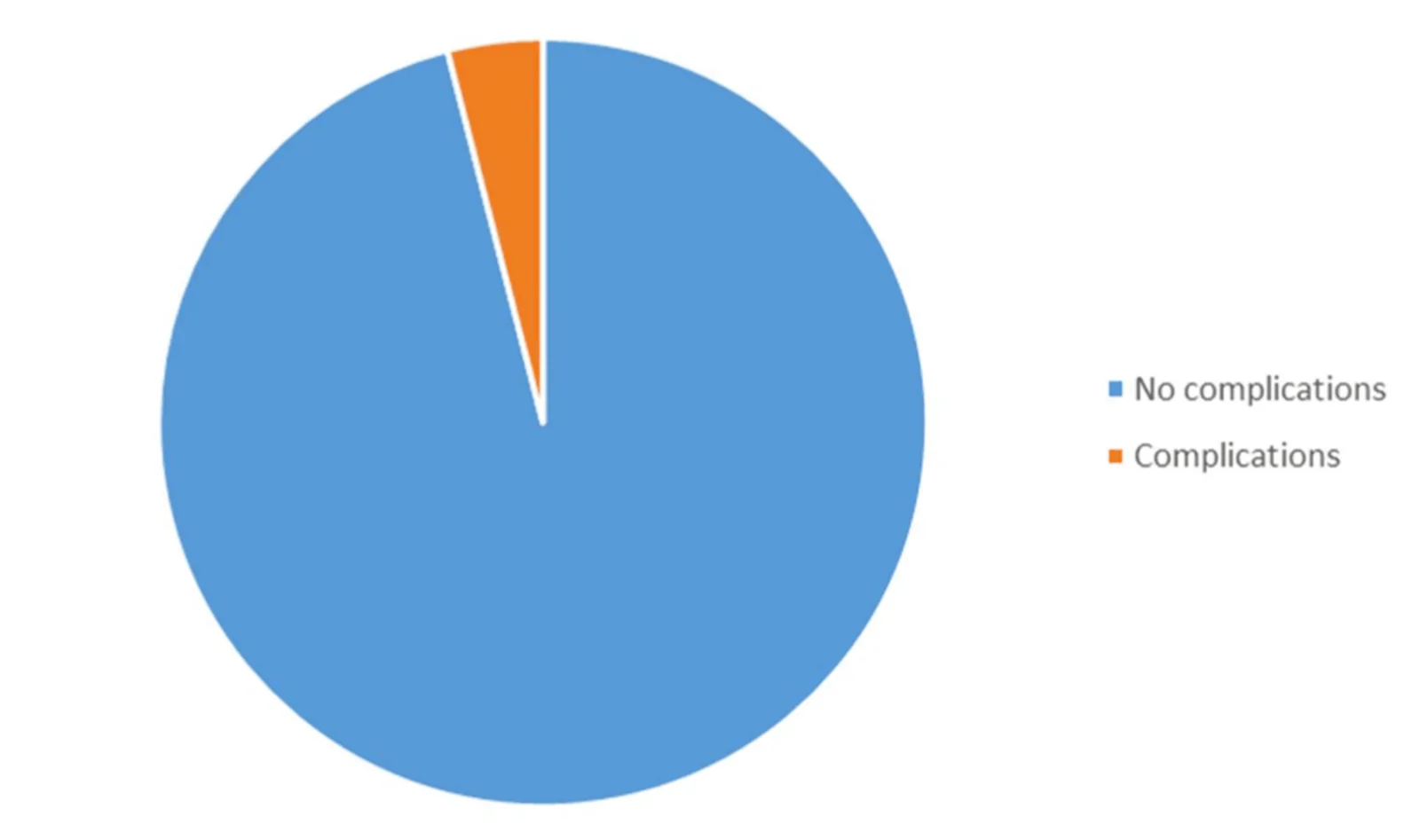
Distribution of postoperative surgical complications among the study participants (n=25)

Functional disability was assessed using the ODI. The mean preoperative ODI score was 48.68 ± 10.85, compared with 17.44 ± 9.70 postoperatively, indicating an improvement in functional status following surgery. The preoperative ODI scores ranged from 30 to 75, while postoperative scores ranged from four to 40 (Tables 7, 8; Figure 9).

**Table 7:** Preoperative Oswestry Disability Index (ODI) scores Values are presented as frequencies and percentages.

Score	Frequency	Percent
30	1	4.0
32	1	4.0
34	2	8.0
38	2	8.0
40	1	4.0
44	2	8.0
46	1	4.0
48	3	12.0
50	1	4.0
52	1	4.0
54	1	4.0
56	3	12.0
58	3	12.0
60	2	8.0
75	1	4.0
Total	25	100.0

**Table 8:** Postoperative Oswestry Disability Index (ODI) scores Values are presented as frequencies and percentages.

Score	Frequency	Percent
4	2	8.0
6	3	8.0
8	2	8.0
10	1	4.0
12	2	8.0
14	1	4.0
16	1	4.0
18	3	12.0
20	5	20.0
22	1	4.0
26	2	8.0
28	1	4.0
40	2	8.0
Total	25	100.0

**Figure 9:**
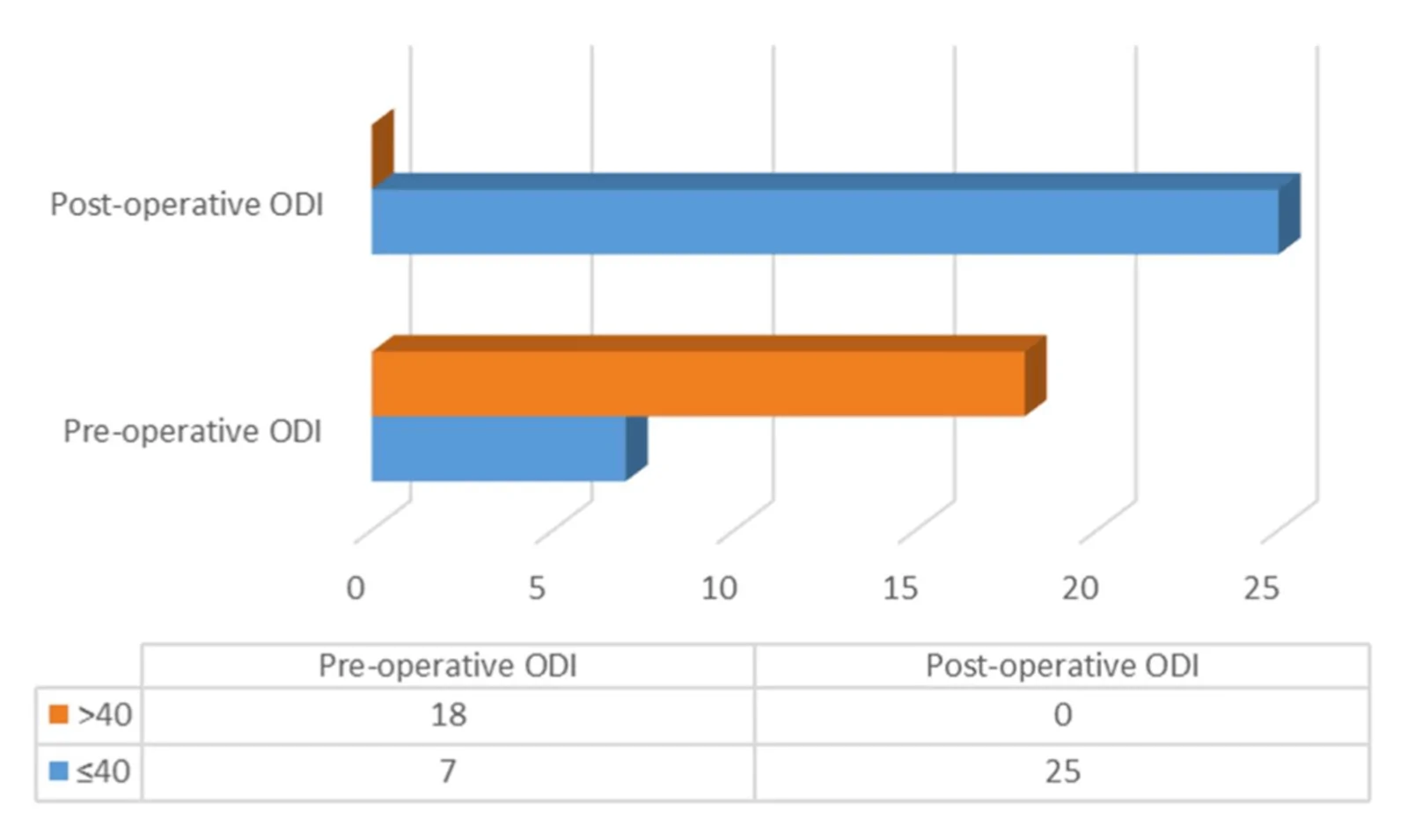
Comparison of pre- and postoperative Oswestry Disability Index (ODI) scores

The median preoperative ODI score was 50, compared with 18 postoperatively (Table 9).

**Table 9:** Descriptive statistics of preoperative and postoperative Oswestry Disability Index (ODI) scores

Statistics	ODI Preoperative	ODI Postoperative
Mean	48.68	17.44
Median	48	20
Mode	48	20
Standard Deviation	10.85	9.70
Range	45	36
Minimum	30	4
Maximum	75	40

Preoperatively, 18 patients (72%) had an ODI score greater than 40, while seven patients (28%) had a score of 40 or below (Tables 10, 11).

**Table 10:** Distribution of preoperative ODI scores by category Oswestry Disability Index (ODI) scores were categorized according to the predefined study thresholds. Values are presented as frequencies and percentages.

≤40	Frequency	Percent
30	1	4.0
32	1	4.0
34	2	8.0
38	2	8.0
40	1	4.0
Total	7	28

**Table 11:** Distribution of postoperative ODI scores by category Oswestry Disability Index (ODI) scores were categorized according to the predefined study thresholds. Values are presented as frequencies and percentages.

ODI scores >40	Frequency	Percent
44	2	8.0
46	1	4.0
48	3	12.0
50	1	4.0
52	1	4.0
54	1	4.0
56	3	12.0
58	3	12.0
60	2	8.0
75	1	4.0
Total	18	72

Postoperatively, all patients had an ODI score of 40 or below, with 23 patients (92%) scoring below 40 and two patients (8%) scoring exactly 40.

A statistically significant difference was observed between the preoperative and postoperative mean ODI scores using the paired samples t-test (Table 12), and when using a one tailed paired samples t-test (Table 13).

**Table 12:** Comparison of preoperative and postoperative Oswestry Disability Index (ODI) scores using a paired-samples t-test Values are presented as mean ± standard deviation. A p-value <0.05 was considered statistically significant.

	Mean	N	Std. Deviation	Correlation	P value	
ODI preoperative	48.6800	25	10.85019	0.412		
ODI postoperative	17.4400	25	9.70429		0.020	0.041

**Table 13:** Comparison of preoperative and postoperative ODI scores using a one-tailed paired-samples t-test A one-tailed paired-samples t-test was performed to assess whether postoperative Oswestry Disability Index (ODI) scores were significantly lower than preoperative scores. A p-value <0.05 was considered statistically significant.

		Std.	Std. Error Mean	95% Confidence Interval of the Difference		t	df	P value
	Mean	Deviation		Lower	Upper			
ODI preoperative - ODI postoperative	31.24000	11.18511	2.23702	26.62301	35.85699	13.965	24	<0.001

Operative time demonstrated considerable variability; however, because operative duration was retrospectively recorded by nursing staff without a standardized protocol and may have included educational activities, these data should be interpreted cautiously. The mean operative time was 96.88 ± 57.50 minutes, and the descriptive statistics are presented in Table 14.

**Table 14:** Operative time Operative time is presented in minutes. Values are summarized using descriptive statistics.

N	Range	Minimum	Maximum	Mean	Std. Deviation
25	226	17	243	96.88	57.524

During the follow-up period, time to return to work was recorded in days following hospital discharge. The distribution of return-to-work duration is presented in Table 15.

**Table 15:** Time to return to work following surgery Values are summarized using descriptive statistics.

N	One day	Two days	Three days	Four days
25	17	5	2	1

## Discussion

In the present study, a total of 25 patients underwent PFEILD at DSFH in Jeddah, Saudi Arabia, between January 2019 and January 2023. The main presenting complaint among the studied patients was LBP associated with lower-limb radiculopathy, which is consistent with the typical clinical presentation of LDH.

The study population demonstrated a nearly equal distribution between the two sexes, with a slight male predominance. This finding is consistent with previous studies that have reported a higher prevalence of LDH among male subjects [3,4,15]. This increased prevalence has been attributed in some studies to occupational and physical factors, particularly occupations involving heavy lifting and repetitive physical loading [3,4,15].

The patients' ages ranged from 20 to 78 years, with a mean age of 42.32 ± 15.79 years. This finding is consistent with the observation that lumbar disc herniation is particularly common during middle adulthood. Satar et al. [6] similarly identified middle age as an important demographic factor associated with LDH.

Most of the participants in the present study were Saudi nationals, which was expected given that the study was conducted at a medical center in Saudi Arabia. Nevertheless, the study population demonstrated considerable ethnic diversity, with approximately eight different nationalities represented, accounting for 32% of the total sample. This diversity provides a degree of representation of the heterogeneous population receiving care at the study center.

Regarding postoperative clinical outcome, the relatively long postoperative follow-up of approximately 24-36 months allowed assessment of the durability of clinical and functional outcomes following PFEILD. The MacNab criteria demonstrated favorable results in the majority of patients. An excellent outcome was reported in 80% (n=20) of patients, while no patient was classified as having a poor outcome. These findings indicate a high rate of satisfactory postoperative clinical improvement following interlaminar PFEILD. Comparable results were reported by Satar et al. [6], who also demonstrated favorable postoperative outcomes following endoscopic lumbar discectomy.

Pain intensity was additionally evaluated using the VAS for leg pain. Preoperatively, 92% (n=23) of patients reported a VAS score greater than 5/10, indicating moderate-to-severe radicular pain in the majority of the study population. Following surgery, a marked reduction in pain intensity was observed, with 96% (n=24) of patients reporting a postoperative VAS score below 5/10. The difference between preoperative and postoperative VAS scores was statistically significant, demonstrating substantial improvement in radicular pain following interlaminar PFEILD.

These findings are in agreement with those reported by Guareschi et al. [29], who evaluated patients using the VAS for leg pain before surgery and 30 days postoperatively. They reported a median preoperative VAS score of seven (range, 6-8), which decreased to three (range, 2-4) postoperatively, with the difference being statistically significant (P<0.001). The consistency between their findings and those of the present study supports the effectiveness of endoscopic lumbar discectomy in reducing postoperative radicular pain.

Functional disability was assessed using the ODI. A substantial improvement was observed following surgery. Preoperatively, 72% (n=18) of the patients had an ODI score greater than 40, whereas none of the patients had an ODI score greater than 40 postoperatively. Furthermore, the difference between the preoperative and postoperative mean ODI scores was statistically significant, indicating significant improvement in functional status following interlaminar PFEILD.

To the best of our knowledge, the finding that none of the patients had an ODI score greater than 40 postoperatively has not been specifically reported in the previously reviewed studies and may represent an important observation of the present study. However, this finding should be interpreted cautiously given the relatively small sample size. Similar postoperative improvement was reported by Hua et al. [30], who demonstrated a reduction in the ODI score from 61.6 (range, 46-84) preoperatively to 16.3 (range, 10-28) one month after surgery. The marked improvement observed in both studies supports the beneficial effect of endoscopic discectomy on postoperative functional recovery.

The findings of the present study are particularly relevant in the context of minimally invasive spinal surgery. Previous studies have highlighted several advantages of percutaneous endoscopic lumbar discectomy (PELD), including reduced tissue trauma, particularly to the paravertebral musculature, preservation of bony structures, reduced nerve-root retraction, and accelerated postoperative rehabilitation [21-23]. These advantages may contribute to shorter hospital stays and earlier functional recovery.

In the present study, 68% (n=17) of patients were discharged within one postoperative day, and no patient required hospitalization beyond approximately four days. No significant postoperative complications requiring prolonged hospitalization were observed. Similar findings regarding the short duration of hospital stay have been reported by Song et al. [15]. The short hospital stay observed in the present study supports the potential role of interlaminar PFEILD as a procedure that may be suitable for selected day-case or short-stay patients. However, the implementation of routine day-case surgery may be influenced by several practical considerations, including institutional protocols, patient selection, postoperative monitoring requirements, and reimbursement policies.

Regarding the anatomical level of disc herniation, L5-S1 was the most frequently affected level in the present study, accounting for 52% (n=13) of cases. The predominance of L5-S1 involvement is consistent with the biomechanical characteristics of the lumbosacral junction. This region represents the transition between the relatively mobile lumbar spine and the more fixed sacrum and is consequently exposed to substantial mechanical loading. Similar findings have been reported by Ali et al. [3], who demonstrated a high frequency of L5-S1 disc involvement.

With respect to the distribution of radiculopathy, right-sided lower-limb symptoms were present in 44% (n=11) of patients, while the remaining patients were equally distributed between left-sided and bilateral symptoms. When radiculopathy was categorized according to laterality, unilateral involvement was more common than bilateral involvement, occurring in 18 and seven patients, respectively. This finding is consistent with the results of Ali et al. [3], who reported unilateral lower-limb radiculopathy in 273 patients compared with bilateral involvement in 195 patients. The predominance of unilateral symptoms in both studies is consistent with the localized nature of most LDHs and their effect on individual nerve roots.

Postoperative complications were uncommon in the present study. Only one patient (4%) developed recurrent disc herniation, whereas 24 patients (96%) experienced no recorded postoperative complications. The reported rates of complications following endoscopic lumbar discectomy vary among studies, as demonstrated in Table 16.

**Table 16:** Reported rates of complications following endoscopic lumbar discectomy The reported rates of complications following endoscopic lumbar discectomy vary among studies.

	Author	Year of Publication	Recurrence Rate
1	Hsu et al. [31]	2013	13.0%
2	Kim et al. [32]	2019	7.0%
3	Ruetten et al. [33]	2008	6.6%
4	Passacantilli et al. [24]	2015	5.0%
5	Yin et al. [34]	2018	4.2%
6	Our Study	2023	4.0%
7	Sencer et al. [35]	2014	3.8%
8	Nasir et al. [36]	2016	2.8%
9	Lee et al. [37]	2016	2.5%
10	Satar et al. [6]	2020	2.0%
11	Hua et al. [30]	2018	1.2%

Differences in reported complication rates may be related to variations in patient selection, surgical technique, surgeon experience, follow-up duration, and definitions of postoperative complications.

One of the most notable findings in the present study was the early return to work. The majority of patients resumed their occupational activities within four days after surgery, suggesting rapid postoperative functional recovery following interlaminar PFEILD. This recovery appeared faster than the seven-day period reported by Tu et al. [46]. Although differences in occupational demands and postoperative rehabilitation protocols may influence the time required to return to work, the early return observed in this study supports the potential advantage of minimally invasive endoscopic surgery in facilitating rapid recovery.

The operative time demonstrated considerable variability, ranging from 17 to 243 minutes, with a non-normal distribution. This variability should be interpreted cautiously because operative time was recorded by nursing staff and the recording process was not performed according to a strict standardized protocol. In some cases, educational sessions involving fellowship trainees may have been included in the recorded operative time. Therefore, the operative-time findings may not accurately reflect the actual surgical time in every case. Standardized prospective recording of operative time would be preferable in future studies.

Overall, the present study demonstrated favorable clinical and functional outcomes following interlaminar PFEILD. Significant improvements were observed in both leg pain, as measured by the VAS, and disability, as measured by the ODI. In addition, the majority of patients achieved an excellent MacNab outcome, while postoperative complications were uncommon. The procedure was also associated with a short hospital stay and early return to work, with 68% of patients discharged within one postoperative day and all patients discharged within approximately four days. These findings highlight the potential benefits of interlaminar PFEILD not only from the patient's perspective, through reduced postoperative pain, disability, and recovery time, but also from a healthcare-system perspective through reduced hospitalization and potentially earlier return to occupational activities.

Nevertheless, the findings should be interpreted in light of the study's limitations, particularly the relatively small sample size and retrospective nature of the study. In addition, the lack of a control or comparison group limits the ability to directly compare interlaminar PFEILD with other surgical approaches. Further prospective studies with larger sample sizes, standardized operative-time recording, longer follow-up, and appropriate comparison groups are warranted to confirm these findings and better define the clinical and economic benefits of interlaminar PFEILD.

Strength

To the best of our knowledge, this is the first study of its kind in Saudi Arabia. Additionally, the study was conducted in advanced health care setting where the documentation and follow up of patients is performed by highly trained medical team. There was no a single missing case during the entire study period.

Limitation

This study does have certain limitations, some of which could be addressed in future research. Firstly, the sample size in our study was relatively small, and the follow-up period was limited in duration. Furthermore, it's worth noting that this study was conducted by a single surgeon in a single location, which could result in a potential bias. The operative time had not have strictly measuring criteria and was measured by different staff members.

## Conclusions

In this cohort of 25 patients, PFEILD was associated with favorable clinical outcomes, including significant improvements in VAS and ODI scores, an 80% excellent outcome according to the modified MacNab criteria, a low recorded complication rate, short hospital stay, and early return to work. However, the findings should be interpreted in the context of the retrospective study design, relatively small sample size, and absence of a control group. Therefore, these results demonstrate favorable outcomes in this cohort but do not establish superiority over other surgical techniques. Larger prospective comparative studies with longer follow-ups are warranted to further evaluate the effectiveness and safety of this technique.
